# *Aedes aegypti* adiponectin receptor-like protein signaling facilitates Zika virus infection

**DOI:** 10.1128/mbio.02433-24

**Published:** 2024-10-07

**Authors:** Tse-Yu Chen, Alejandro Marín-López, Hamidah Raduwan, Erol Fikrig

**Affiliations:** 1 Section of Infectious Diseases, Department of Internal Medicine, School of Medicine, Yale University, New Haven, Connecticut, USA; The Ohio State University, Columbus, Ohio, USA; The University of Hong Kong, Pokfulam, Hong Kong

**Keywords:** mosquito, Zika virus, adiponectin receptor, trypsin, midgut, *Aedes aegypti*

## Abstract

The *Aedes aegypti* mosquito plays a critical role in the transmission of viral diseases, including Zika virus, which poses significant public health challenges. Understanding the complex interactions between mosquitoes and viruses is paramount for the development of effective control strategies. In this study, we demonstrate that silencing the *A. aegypti* adiponectin receptor-like protein (AaARLP) results in a reduction of Zika virus infection. Transcriptomic analysis identified alterations in several trypsin genes and further revealed that *AaARLP*-knockdown mosquitoes had diminished trypsin activity. Moreover, silencing of selected *trypsins* resulted in a similar delay in Zika virus infection in mosquitoes, further highlighting the connection between the AaARLP and trypsin. Overall, our findings demonstrate that AaARLP signaling is important for Zika virus infection of *A. aegypti*.

**IMPORTANCE:**

Arboviruses pose a significant threat to public health, with mosquitoes, especially *Aedes aegypti*, being a major vector for their transmission. Gaining insight into the complex interaction between mosquitoes and viruses is essential to build successful control strategies. In this study, we identified a novel pathway connecting the *A. aegypti* adiponectin receptor-like protein and its association with trypsin, key enzymes involved in blood digestion. Furthermore, we demonstrated the significance of signaling via the adiponectin receptor-like protein in virus infection within the mosquito. Together, our discoveries illuminate mosquito metabolic pathways essential in viral infection.

## INTRODUCTION

Zika virus (ZIKV) is a flavivirus family with a 10.8 kb single-stranded RNA genome. It was first isolated in Uganda in 1947 and caused the ZIKV epidemic in the Americas between 2015 and 2016 (1). ZIKV is primarily transmitted by *Aedes* mosquitoes, especially *Aedes aegypti*, and can cause Zika fever as well as microcephaly and severe brain malformations in newborns (2). Evidence of mosquito-transmitted ZIKV infection has been reported in 89 countries and territories (3). Considering the expanding range of vectors and the prevalence of global travel (4), the potential risk of ZIKV infection to public health remains high. However, no specific treatments or vaccines have been approved for clinical use (5), emphasizing the urgent need to develop new strategies to halt the transmission cycle.

Numerous innate immune responses are triggered to limit viral replication in mosquitoes (6). Beyond these immune pathways, a substantial number of mosquito genes unrelated to immunity frequently demonstrate altered transcription levels in response to virus infection, and some may exert an influence on virus replication in mosquitoes (7). Furthermore, metabolic changes, involving complicated chemical reactions in mosquitoes, undergo alterations crucial for general physiological functions and organismal survival in response to virus infection (8).

The adiponectin receptor in mammals is activated by adiponectin (9, 10) and other synthetic small molecules (11, 12). Adiponectin is secreted by adipocytes and maintained at a high concentration in blood. While the adiponectin receptor is known to play significant roles in regulating metabolism, insulin sensitivity, and inflammation across different species (13, 14), its function in *A. aegypti* remains unclear. The adiponectin pathway has shown associations between several arthropods and pathogens. In *Anopheles gambiae*, activation of the adiponectin receptor-like protein has been reported to decrease *Plasmodium* infection (15). Conversely, in *Ixodes scapularis*, the adiponectin receptor-like protein pathway triggers phospholipid metabolism that contributes to *Borrelia burgdorferi* colonization (16). Crosstalk between adiponectin and insulin signaling pathways occurs at multiple tissues in mammals (17), and evidence has shown that the activated insulin pathway in *A. aegypti* triggers the JAK/STAT and RNA interference (RNAi) pathways, serving as an antiviral response (18).

In this study, we characterized an *A. aegypti* adiponectin receptor-like protein (AaARLP) from *A. aegypti*. RNAi studies demonstrated that AaARLP is associated with ZIKV infection in the mosquito midgut. Furthermore, transcriptomic and functional experiments revealed that AaARLP is connected with trypsin activity, a crucial enzyme for mosquito blood meal digestion (19). Taken together, our findings identify the role of the AaARLP pathway in *A. aegypti*, emphasizing the metabolic pathways involved in ZIKV infection in the vector.

## RESULTS

### Identification of an adiponectin receptor-like protein in *A. aegypti*

**FIG 1:**
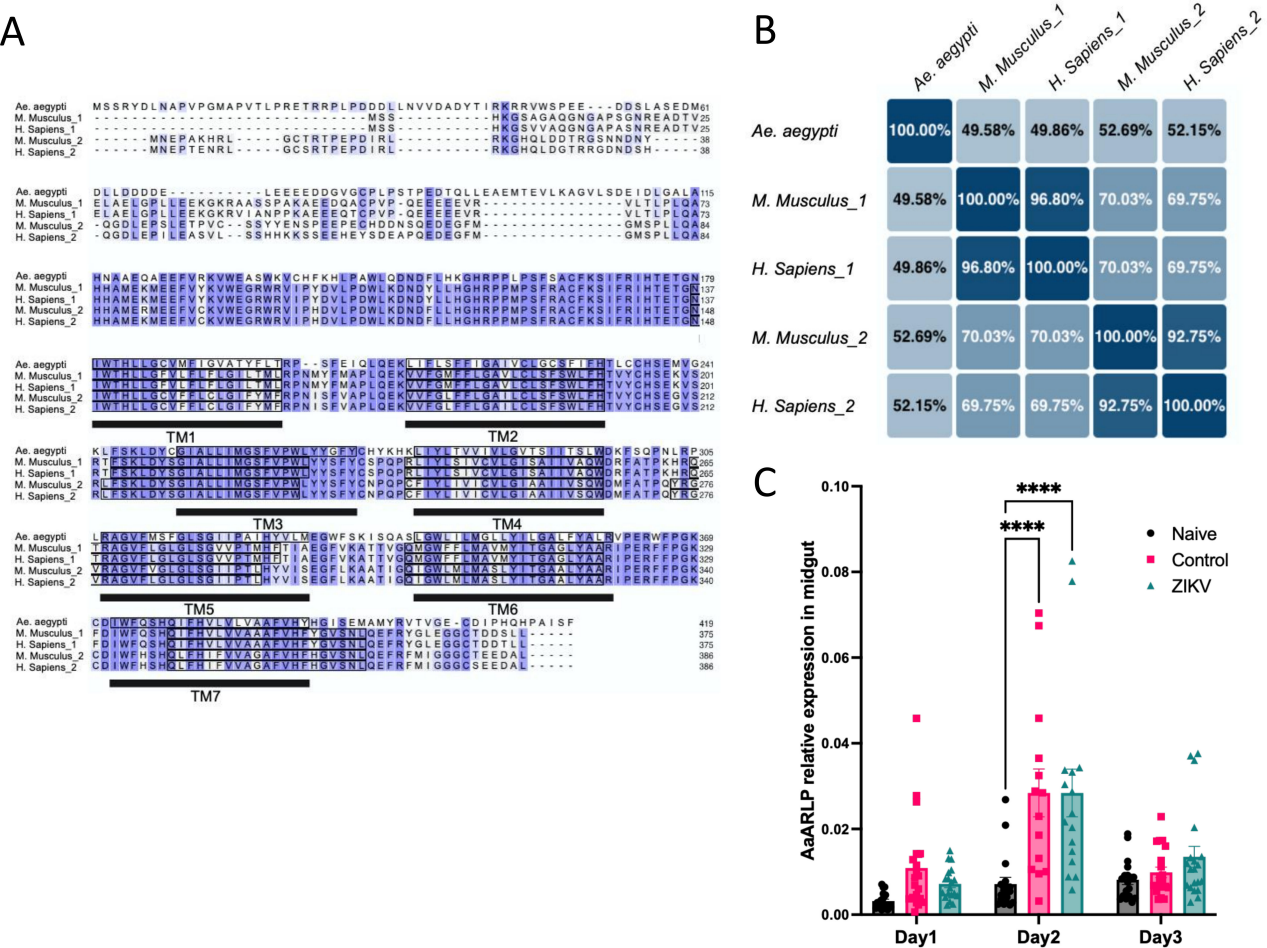
Comparison of the sequence of the adiponectin receptor-like protein (AaARLP) in *A. aegypti* with mammalian adiponectin receptors 1 and 2. (A) Sequence alignment with marked seven transmembrane domains. (B) The percent identity matrix depicts the comparison of adiponectin receptors across different species. The percentage numbers indicate the degree of similarity between the protein sequences. (C) AaARLP transcript in the *A. aegypti* midgut among Naïve, Control, and ZIKV groups at different time points. Each dot represents one midgut sample, with error bars indicating the standard error of the mean (SEM). Day 1: Naïve *N* = 19, Control *N* = 20, and ZIKV *N* = 18; Day 2: Naïve *N* = 18, Control *N* = 14, and ZIKV *N* = 16; Day 3: Naïve *N* = 18, Control *N* = 20, and ZIKV *N* = 20. ****P-value < 0.0001.

The AaARLP in *A. aegypti* (AAEL014717) has been documented in both the National Center for Biotechnology Information (NCBI) and Vector Base databases (https://vectorbase.org/). AaARLP possesses a conserved domain known as Hemolysin-III (HlyIII), which is commonly found in functional receptors, with seven transmembrane domains. Unlike mammalian adiponectin receptors, AaARLP does not have an isoform. Comparative alignment results were obtained for AaARLP, adiponectin receptors 1 and 2 of *Homo sapiens* and *Mus musculus* (Fig. 1A). The results showed substantial similarity, particularly in the region where the HlyIII domain was located. Higher variability was noticed in the N-terminal region. The identity percentages revealed that AaARLP shares 49% identity with the adiponectin receptor 1 of *M. musculus*, 53% with adiponectin receptor 2 of *M. musculus*, 50% with adiponectin receptor 1 of *H. sapiens*, and 52% with adiponectin receptor 2 of *H. sapiens* (Fig. 1B).

To better understand how *AaARLP* may be stimulated in the midgut, mosquitoes were categorized into three groups: Naïve, Control, and ZIKV. Female mosquitoes were fed sugar water (Naïve), AG129 mouse (Control), or ZIKV-infected AG129 mouse [4.6 ± 0.6 log plaque-forming unit (PFU)/mL] (ZIKV), respectively. Subsequently, the midguts of these mosquitoes were collected to analyze the expression levels of the *AaARLP* transcript (Day 1: Naïve *N* = 19, Control *N* = 20, and ZIKV *N* = 18; Day 2: Naïve *N* = 18, Control *N* = 14, and ZIKV *N* = 16; Day 3: Naïve *N* = 18, Control *N* = 20, and ZIKV *N* = 20) (Fig. 1C) and to confirm the ZIKV infection status (Fig. S1). The transcript expression levels revealed that both the control and ZIKV groups had higher expression levels at the 2-day post-feeding stage compared to the naïve group (*P*-value < 0.0001). However, there were no significant differences in *AaARLP* expression levels among the three groups on the 1- and 3-day post-feeding stages, suggesting that day 2 might be the crucial time for evaluating AaARLP.

### Silencing *AaARLP* impacts the virus infection in both Aag-2 cells and in *Ae. aegypti*

**FIG 2:**
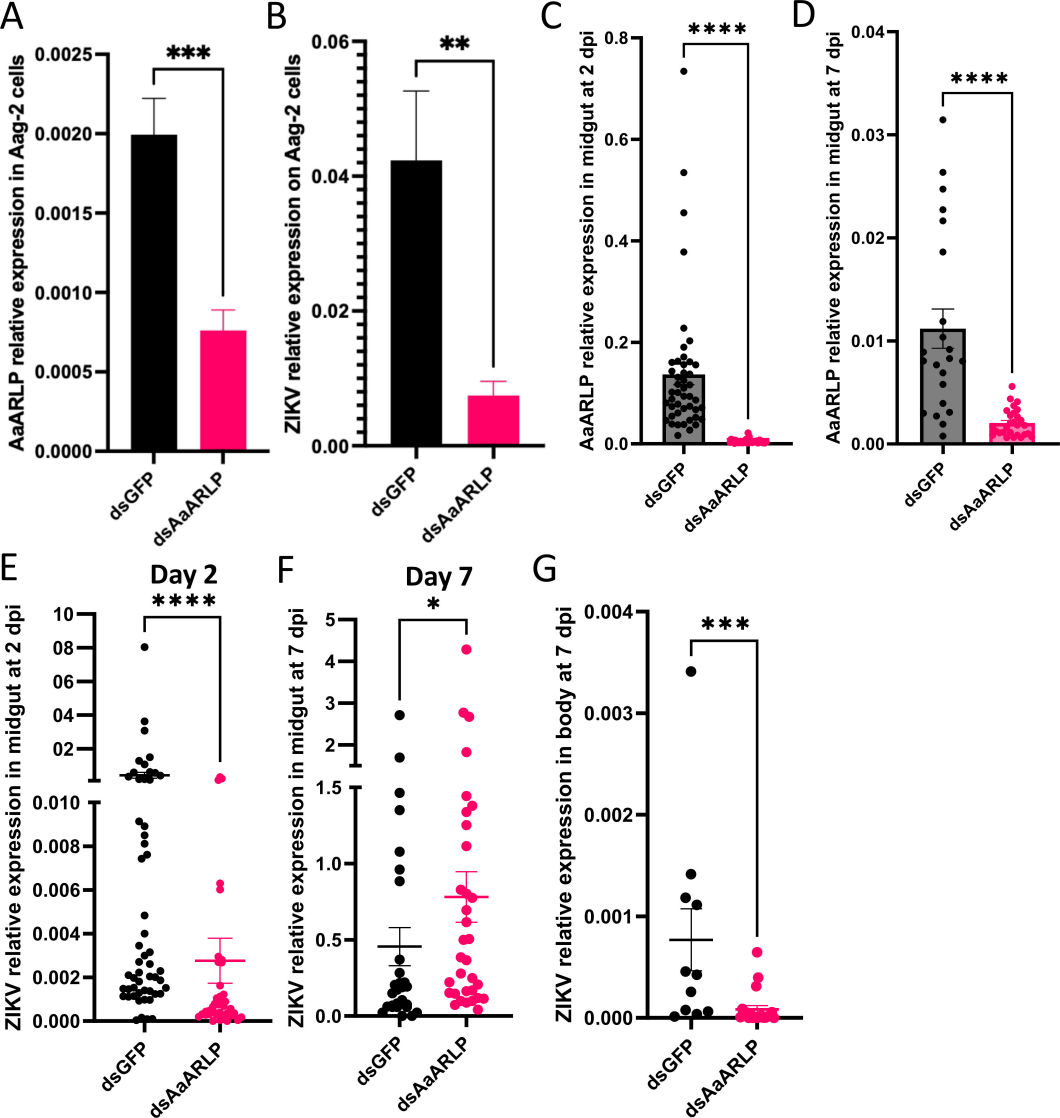
Silencing of adiponectin receptor-like protein (AaARLP) impacted ZIKV RNA copy numbers in Aag-2 cells and *A. aegypti*. (A) Relative expression levels of *AaARLP* and (B) ZIKV between dsGFP and ds*AaARLP* at 2 days post-infection in Aag-2 cells. The bars indicate the mean with standard error of the mean (SEM). Both dsGFP and dsAaARLP *N* = 8. Relative expression levels of *AaARLP* between dsGFP and ds*AaARLP A. aegypti* midgut (C) 2 days and (D) 7 days post ZIKV infection. The ZIKV RNA copy numbers between dsGFP and ds*AaARLP A. aegypti* (E) 2 days in midgut, (F) 7 days in midgut, and (G) 7 days in the body post ZIKV infection. Each dot represents one midgut sample, with error bars indicating the SEM. Day 2 midgut: dsGFP *N* = 54, dsAaARLP *N* = 39; Day 7 midgut: dsGFP *N* = 46, dsAaARLP *N* = 54; Day 7 body: dsGFP *N* = 22, dsAaARLP *N* = 32. *P-value < 0.05, **P-value < 0.01, ***P-value < 0.001, and ****P-value < 0.0001.

AaARLP was silenced in an *A. aegypti* cell line (Aag-2) to explore its role in ZIKV infection. After transfecting Aag-2 cells with dsRNA for 3 days, the cells were infected with 0.1 MOI of ZIKV and then collected 2 days post-infection. The silencing process exhibited an efficiency of 62%, resulting in a significant decrease in *AaARLP* expression within cells (Fig. 2A). The virus RNA copy number was significantly decreased following *AaARLP* silencing in Aag-2 cells (*P*-value = 0.001) (Fig. 2B) indicating a possible connection between AaARLP and ZIKV.

Female mosquitoes aged 4–7 days were subjected to microinjection with targeted dsRNA to induce *AaARLP* knockdown. Following a 3-day interval, both the dsGFP and ds*AaARLP* groups were fed on the same ZIKV-infected AG129 mouse (5.1 ± 0.2 log PFU/mL). The midguts were collected after 2- and 7-day post-infection and the rest of the bodies were also collected at 7-day post-infection to examine the dissemination. The *AaARLP* expression levels were assessed to verify the efficiency of knockdown using samples from the midguts collected at 2- and 7-day post-infection. The knockdown efficiency of *AaARLP* was 97% in the midgut samples from the 2 day post-infection (*P*-value < 0.0001) (Fig. 2C). At 7 days post-infection, the knockdown efficiency of *AaARLP* remained at 82% in the midgut samples (*P*-value < 0.0001) (Fig. 2D).

In the *AaARLP*-silenced group (ds*AaARLP*, *N* = 54), the ZIKV RNA copy number within the midgut was significantly lower than that in the control group (dsGFP, *N* = 41, *P*-value < 0.0001) at 2 day post-infection (Fig. 2E). At the 7-day post-infection, no disparity in infection rate (positive ZIKV infected midgut/total midgut tested) was observed between the dsGFP group (22/46, 48%) and the ds*AaARLP* group (32/54, 57%). Nonetheless, within the successfully infected midguts, the *AaARLP*-silencing group displayed a notably higher viral titer than the dsGFP-injected group (*P*-value = 0.027) (Fig. 2F). While the body dissemination rate (positive ZIKV infected body/body from the positive midgut sample) exhibited no distinction between the two groups (dsGFP: 11/22, 50%; ds*AaARLP*: 19/32, 59%), the ds*AaARLP* group, which showed positive infection, displayed a significant reduction in ZIKV levels compared to the dsGFP group (*P*-value < 0.001) (Fig. 2G). Silencing *AaARLP* reduced viral RNA levels at early stages. Conversely, at later stages, the midgut had increased viral RNA levels, while the rest of the body showed decreased levels in the ds*AaARLP* group. Overall, the results indicate that *AaARLP* knockdown hinders ZIKV infection, slowing down the virus’s infection progress and spread from the midgut.

### RNA-seq at 2- and 7-days post-infection with *AaARLP* silencing in the midgut

To investigate the mechanisms underlying the connection between AaARLP and ZIKV, three individual mosquito midguts from the dsGFP group and three from the ds*AaARLP* group, collected at 2- or 7-days post-infection, underwent RNA-seq analysis to elucidate differences in their transcriptomes. Under the conditions of a *P*-value < 0.05, false discovery rate ≤ 0.1 and a fold change > 2, the only gene showing a difference between dsGFP and ds*AaARLP* at day 2 is *AaARLP*, which is downregulated, confirming the silencing efficiency in the samples. However, no other significant genes were detected in the day 2 RNA-seq results, suggesting that AaARLP might not impact the transcriptome at the early time point.

**FIG 3:**
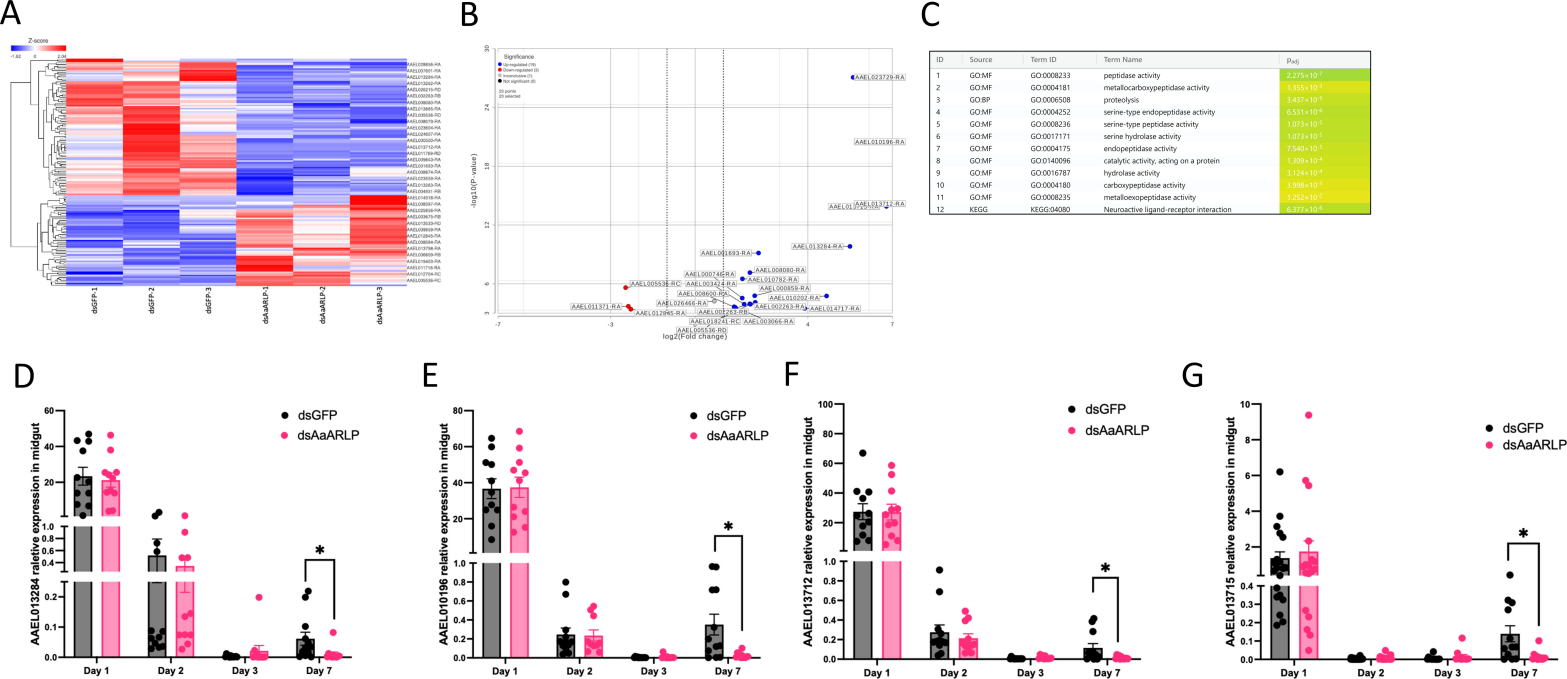
RNA-sequencing results and validation from the *A. aegypti* midgut between dsGFP and ds*AaARLP* 7 days post-infection. (A) Heatmap displayed in a grid where each row represents a gene, and each column represents a sample. (B) Volcano plot shows statistical significance versus fold change. (C) The functional enrichment analysis from the significant transcripts. The relative expression level of trypsin genes (D) AAEL013284, (E) AAEL010196, (F) AAEL013712, and (G) AAEL013715 between dsGFP and ds*AaARLP* in the *A. aegypti* midgut at 1-, 2-, 3-, and 7-days post-blood feeding. Each dot represents one midgut sample, with error bars indicating the standard error of the mean (SEM). Day 1: dsGFP *N* = 20, dsAaARLP *N* = 19; Day 2: dsGFP *N* = 24, dsAaARLP *N* = 21; Day 3: dsGFP *N* = 12, dsAaARLP *N* = 11; Day 7: dsGFP *N* = 12, dsAaARLP *N* = 12. *P-value < 0.05.

From the analysis of 7-days post-infection, 22 genes exhibited significant differences between the midgut groups of dsGFP and ds*AaARLP* under the conditions of a *P*-value < 0.05, false discovery rate ≤ 0.1 and a fold change > 2, with 3 transcripts showing an increase and 19 transcripts downregulated in the ds*AaARLP* group (Fig. 3A and B; Table S1). The expression of *AaARLP* was significantly lower in the ds*AaARLP* group, as expected, highlighting the efficiency of silencing. Functional enrichment analysis revealed a statistically significant enrichment of genes associated with peptidase activity and proteolysis (Fig. 3C).

For validation, the late trypsin 1 (AAEL013284), trypsin (AAEL010196, AAEL013715), and trypsin 5G1 precursor (AAEL013712) were chosen, and significant differences between dsGFP and ds*AaARLP* midguts at 7 days post-infection were confirmed (AAEL013284, *P*-value = 0.01; AAEL010196, *P*-value = 0.017; AAEL013712, *P*-value = 0.045; AAEL013715, *P*-value = 0.024) (Fig. 3D through G). However, no differences were observed between dsGFP and ds*AaARLP* in trypsin transcript levels at days 1, 2, and 3 post-infections in the midgut (Fig. 3D through G), confirming the RNA-seq data from day 2.

### Trypsin activity decreased following the silencing of *AaARLP*

**FIG 4:**
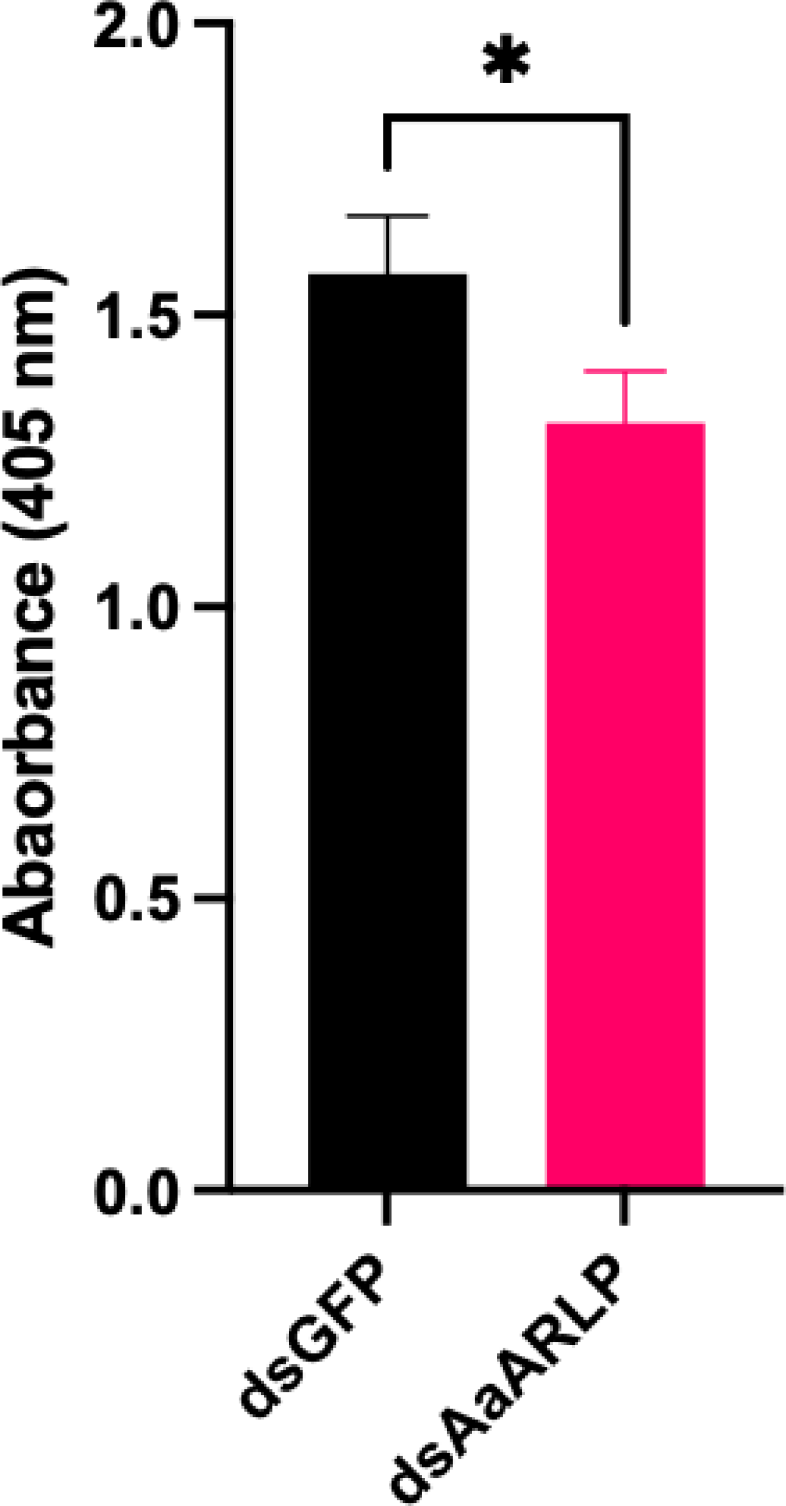
Inhibition of *A. aegypti* midgut trypsin activity after silencing of *adiponectin receptor-like protein* (*AaARLP*). Trypsin *in vivo* enzyme activity in dsGFP and ds*AaARLP* mosquito midguts 24 h post-blood feeding. Error bars represent the standard error of the mean (SEM). A total of 8 groups of dsGFP and 11 groups of ds*AaARLP* were collected, with each group containing 10 midguts. The dsGFP *N* = 8 and dsAaARLP *N* = 11. *P-value < 0.05.

Trypsin transcript levels were unaffected by AaARLP at early time points post-infection but showed changes at later stages. To understand the connection between AaARLP and trypsin in the mosquito midgut, a trypsin activity assay was conducted to assess enzyme activity. Mosquito midguts were collected 24 h post-blood feeding, and absorbance was compared between dsGFP and ds*AaARLP* samples. The absorbance was found to be significantly lower in the ds*AaARLP* midguts (*P*-value = 0.041) (Fig. 4), suggesting that AaARLP influences trypsin activity post-blood feeding.

### Silencing trypsin genes impacts virus infection in *A. aegypti*

**FIG 5:**
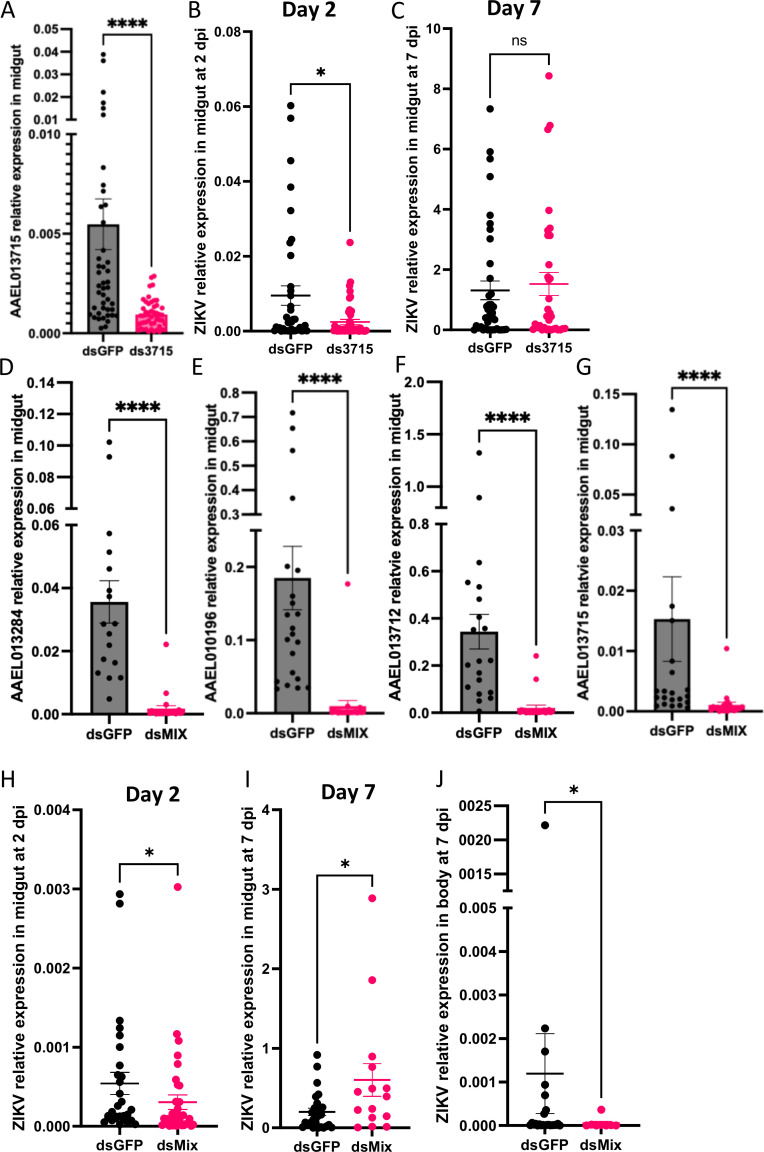
Impact of trypsin silencing on ZIKV RNA copy numbers in *A. aegypti*. (A) Relative expression levels of trypsin (AAEL013715) between dsGFP and ds3715 at 2 days post-infection in the *A. aegypti* midgut. (B) ZIKV RNA copy numbers between dsGFP and ds3715 at 2 days and (C) 7 days post-infection in the *A. aegypti* midgut. Day 2: dsGFP *N* = 39, ds3715 *N* = 47; Day 7: dsGFP *N* = 39, ds3715 *N* = 33. Relative expression levels of trypsin genes (D) AAEL013284, (E) AAEL010196, (F) AAEL013712, and (G) AAEL013715 between dsGFP and dsMIX in the *A. aegypti* midgut at 2 days post-infection. ZIKV RNA copy numbers between dsGFP and dsMIX *A. aegypti* at (H) 2 days in midgut, (I) 7 days in midgut, and (J) 7 days in the body post-ZIKV infection. Day 2 midgut: dsGFP N 29, dsMix *N* = 37; Day 7 midgut: dsGFP *N* = 42, dsMix *N* = 43; Day 7 body: dsGFP *N* = 27, dsMix *N* = 15. Each dot represents one midgut sample, with error bars indicating the standard error of the mean (SEM). *P-value < 0.05 and ****P-value < 0.0001.

To determine whether trypsin affects ZIKV infection, female mosquitoes aged 4–7 days were subjected to microinjection with targeted dsRNA to silence trypsin genes identified from the RNA-seq analysis (ds3284, AAEL013284; ds0196, AAEL010196; ds3712, AAEL013712; ds3715, AAEL013715). Following a 3-day interval, all groups were fed on the same ZIKV-infected AG129 mouse (4.9 ± 0.9 log PFU/mL). The midguts were collected 2- and 7-days post-infection, while the remaining bodies were collected exclusively at the 7-day post-infection time point to assess dissemination. To verify the efficiency of knockdown, all the four trypsin expression levels were assessed using samples from the midguts collected at 2 days post-infection. The knockdown efficiency was 99% in ds3284 (*P*-value < 0.0001) (Fig. S2A), 99% in ds0196 (*P*-value < 0.0001) (Fig. S2B), 98% in ds3712 (*P*-value < 0.0001) (Fig. S2C), and 83% in ds3715 (*P*-value < 0.0001) (Fig. 5A) in the midgut samples.

In the ds3715 group (*N* = 47), the ZIKV RNA copy number within the midgut was significantly lower than that in the control group (*N* = 39, *P*-value = 0.0173) at 2 days post-infection (Fig. 5B). However, no virus difference was noted in the other three trypsin-silencing groups at 2 days post-infection (Fig. S2D). At 7 days post-infection, the ZIKV RNA copy number showed no difference in either trypsin-silencing midgut or body (Fig. 5C; Fig. S2E and F).

As single trypsin knockdowns did not replicate the effects of *AaARLP* silencing, the four trypsin dsRNAs were combined into a cocktail and microinjected into mosquitoes to investigate the function of multiple trypsins identified from the RNA-seq (dsMIX). After 3-day, both dsGFP and dsMIX groups were fed on the same ZIKV-infected AG129 mouse (4.3 ± 0.0 log PFU/mL), and samples were collected at the same time points as described earlier. To assess the efficiency of knockdown, all four trypsin expression levels were examined using samples from the midguts collected at 2 days post-infection in both dsGFP and dsMIX groups. The knockdown efficiency was 95% in ds3284 (*P*-value < 0.0001) (Fig. 5D), 94% in ds0196 (*P*-value < 0.0001) (Fig. 5E), 94% in ds3712 (*P*-value < 0.0001) (Fig. 5F), and 93% in ds3715 (*P*-value < 0.0001) (Fig. 5G).

In the dsMIX group (*N* = 37), the ZIKV RNA copy number within the midgut significantly decreased compared to the dsGFP group (*N* = 29, *P*-value = 0.0283) at 2 days post-infection (Fig. 5H). By the 7th day post-infection, a significant difference in infection rates emerged between the dsGFP group (27/42, 64%) and the dsMIX group (15/43, 35%; *P*-value = 0.0093). However, within the infected midguts, the dsMIX group exhibited a markedly higher viral number than the dsGFP-injected group (*P*-value = 0.0451) (Fig. 5I). While the body dissemination rate showed a distinction between the two groups (dsGFP: 24/27, 89%; dsMIX: 8/15, 53%; *P*-value = 0.02), the dsMIX group, that displays positive infection demonstrated a significant reduction in ZIKV levels compared to the dsGFP group (*P*-value = 0.0258) (Fig. 2F). These results indicate that the dsMIX delays ZIKV infection progression, further confirming the connection between AaARLP and trypsin, and demonstrating the impact of this pathway on the virus in mosquitoes.

## DISCUSSION

Adiponectin receptor-mediated pathways are known to regulate glucose levels and facilitate fatty acid breakdown (20), demonstrating its importance in various pathogen–vector interactions (15, 16). In this study, we provide evidence that the AaARLP pathway in *A. aegypti* is crucial for virus infection. Furthermore, the downstream signaling of the AaARLP pathway is connected to trypsin activity, representing a significant contribution to our understanding of mosquito metabolic pathways and their interaction with virus infection.

AaARLP in *A. aegypti* contains HlyIII, which possesses seven transmembrane domains known to encode functional receptors with a broad range of apparent ligand specificities (21). This suggests that AaARLP could function as a receptor on the cell membrane, like the adiponectin receptors in mammals (Fig. 1A and B). The similarity between AaARLP and mammalian adiponectin receptors is approximately 50%, with most differences located in the cytoplasmic N-terminal domain. This domain is crucial for adiponectin downstream signaling in mammals (22), and the sequence differences in mosquitoes might lead to different functions or signaling pathways for AaARLP. The upregulation of the *AaARLP* transcript after a blood meal in the midgut indicates a relationship between metabolic activity and AaARLP, possibly due to the blood stimulation (23) (Fig. 1C).

Silencing *AaARLP* resulted in a decrease in virus RNA copy numbers at the early time points in both *in vitro* (Fig. 2B) and *in vivo* (Fig. 2E) experiments. However, at the late time point, the virus RNA copy numbers in the midgut were higher (Fig. 2F) but exhibited lower virus RNA copy numbers in the body in the ds*AaARLP* group (Fig. 2G). Taken together, these findings suggest that *AaARLP* knockdown delays the progression of ZIKV infection, prolonging the time required for the virus to successfully replicate and escape from the midgut. This result contrasts with data from human A549 epithelial cells, where the activated adiponectin pathway exhibited an antiviral response to ZIKV (24). However, it is important to consider the tissue-specific signaling pathways of the adiponectin receptor-mediated pathways (17), and the differences between mosquitoes and humans. Additionally, A549 cells were isolated from the lung, whereas mosquito midgut is the primary site of infection, contributing to the observed differences in the reaction to ZIKV infection. Considering AaARLP role as a receptor, the delay in virus infection may result from alterations in downstream signaling. RNA-seq analysis was performed at both early and late time points to identify potential targets. While no transcript differences were observed early on, the RNA-seq data revealed a decrease in transcript levels of several digestion enzymes at later time point, including trypsin, serine-type endopeptidase, and carboxypeptidase, after silencing *AaARLP* (Fig. 3A and B). Functional analysis also showed that these transcripts are associated with peptidase activity (Fig. 3C), which strongly suggests that AaARLP is highly associated with enzymes related to metabolism.

Trypsin in mosquito is characterized in two groups, early and late trypsin (19). In the initial 4–6 h post-blood meal, there are trace amounts of early trypsin (25, 26). Subsequently, the second phase, occurring between 8 and 36 h after blood feeding, is characterized by a substantial presence of late trypsin (27, 28). The late trypsin transcript peaks 24 h post-feeding and gradually decreases (29). All four trypsins identified from the RNA-seq belong to the late trypsin family and exhibit a consistent pattern of transcript expression across various time points. Although the differences in expression level between the control and ds*AaARLP* groups were noted at day 7 post-infection, no distinctions were observed at day 1–3 (Fig. 3D through G), suggesting AaARLP might not regulate the gene expression of trypsin in the early phase of blood ingestion, which could be triggered by early trypsin digestion products (19). However, we noticed the trypsin activity was decreased in the ds*AaARLP* midgut 24 h post-blood feeding (Fig. 4), indicating a connection between AaARLP and trypsin at the protein level.

Different trypsins have been reported to alter the mosquito susceptibility to virus (30–32). One trypsin (AAEL013715) demonstrated an impact at the early time point of ZIKV infection; however, no difference was observed at the later time point, consistent with the results of the other three trypsins tested (Fig. 5A through C; Fig. S2). These findings align with a previous study, where both ds3715 and ds0196 did not alter the DENV titer in the *A. aegypti* midgut at 7 days post-infection (32). However, when all four trypsins were combined, similar effects were observed as with AaARLP, resulting in the delay of ZIKV infection (Fig. 5I through K).

Previous studies have shown that the application of soybean trypsin inhibitor (STI) into the infectious blood meal slowed DENV replication in the midgut and decreased dissemination to other tissues (30). Moreover, silencing different combinations of trypsins led to an increased DENV titer in the midgut at 7 days post-infection (32). The data suggest that trypsin is the factor causing the delay of virus replication and has shown similar outcomes across different viruses and *A. aegypti* populations, possibly through a nutritional effect (30). Furthermore, the similar outcome between ds*AaARLP* and dsMIX provides additional evidence of the connection between AaARLP and trypsin. Although trypsin plays a crucial role in mosquito blood digestion, the related signaling pathways remain unclear. AaARLP could be one of the receptors influencing trypsin activity. However, mosquitoes lack homologs of some crucial downstream effectors in the mammalian adiponectin pathway, such as adaptor protein, phosphotyrosine interacting with PH domain, and leucine zipper 1 (APPL1) (33). Combined with the diverse N-terminal domain of AaARLP, it is possible that the signaling pathway differs between mammals and mosquitoes. Further experiments are needed to identify the specific signal transducers involved.

Silencing AaARLP did not have as pronounced an effect on viral infection as observed with other immune-related factors (34), suggesting that its pathway is not directly linked to the virus but instead affects secondary factors, such as trypsin enzymatic activity and nutritional processes (30). The enzyme activity assay revealed a significant reduction in trypsin activity, though the enzyme remained functional, indicating that AaARLP is one of multiple pathways involved in trypsin regulation (Fig. 4). This suggests that additional pathways may intersect with trypsin, which could explain why the AaARLP pathway alone did not exert a strong impact on viral infection. Our findings reveal the AaARLP-trypsin pathway, and future studies should focus on identifying other pathways regulating trypsin and potential crosstalk between these pathways.

Our results on AaARLP reveal a part of the complex physiological networks in mosquitoes and provide a possible target for control strategy development. Vectorial capacity describes the potential of a vector to transmit a pathogen (35). The function of AaARLP is crucial for the virus infection process, highlighting key factors in vectorial capacity, such as the pathogen’s extrinsic incubation period and vector competence (36). Developing small molecules targeting AaARLP could potentially serve as a mosquito vaccine to interrupt vectorial capacity (18, 37, 38). While limitations in mosquito research, such as the availability of antibodies and suitable agonists/antagonists, exist, we were able to achieve significant findings through alternative approaches. We focused on the mosquito midgut due to its relationship with virus infection (39, 40). However, the development of antibodies could provide further insights into AaARLP localization in different tissues. Additionally, generating antibodies targeting key factors from various pathways, such as AMP-activated protein kinase (AMPK), a downstream component of the adiponectin receptor pathway in mammals (17), could reveal important details of the AaARLP signaling pathway in mosquito. Once this information is available, it would be valuable to explore whether AaARLP plays distinct roles in other mosquito tissues, such as the fat body. Despite the limitation, we have successfully established the AaARLP-trypsin pathway, showcasing the potential for further discoveries in mosquito biology.

**FIG 6:**
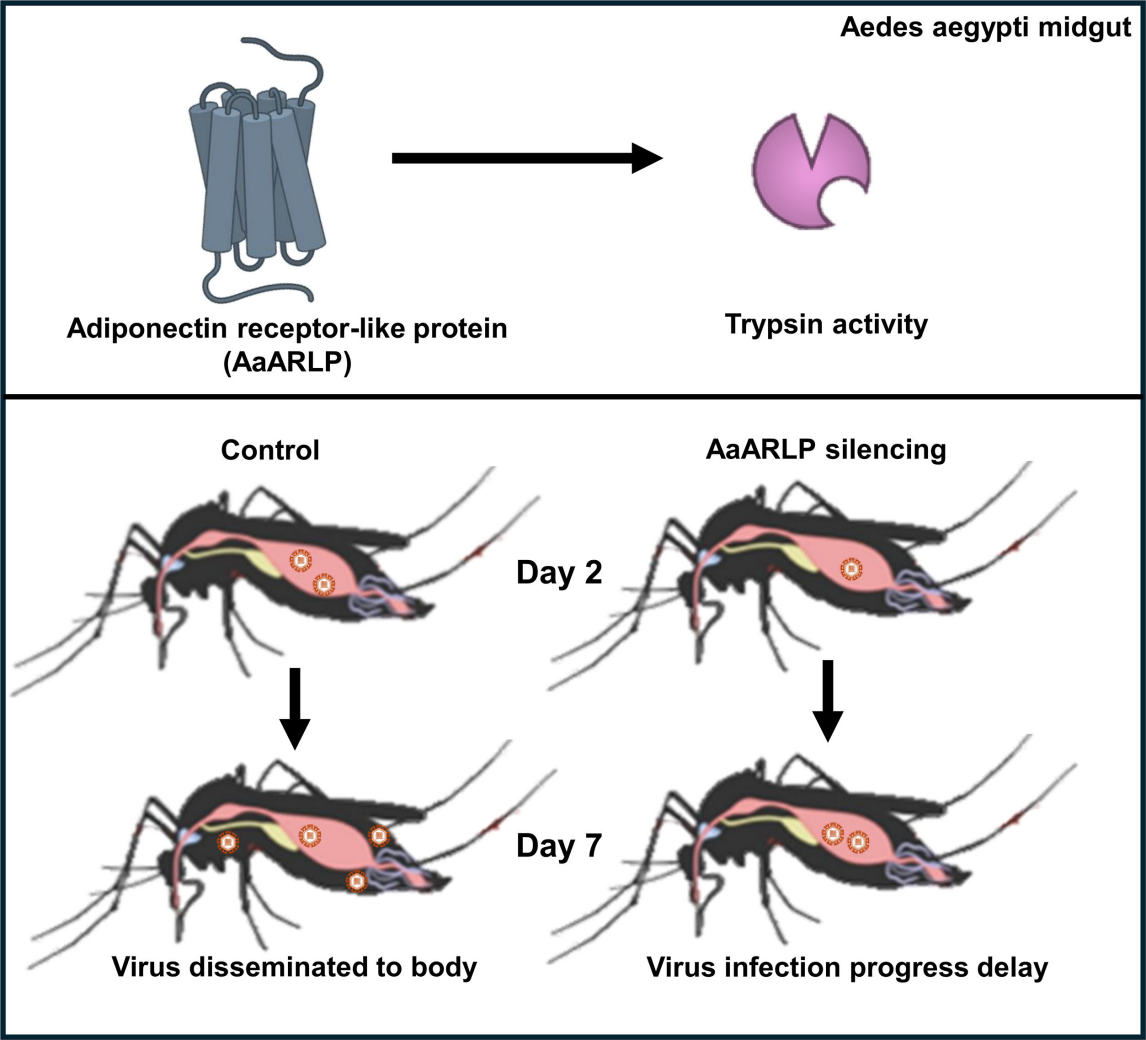
Model of adiponectin receptor-like protein in delaying ZIKV infection. The adiponectin receptor-like protein affects trypsin enzymatic activity early in the infection process, significantly influencing both the progression of the virus infection and the timing of dissemination.

In summary, we have identified a novel pathway involving an AaARLP that plays a crucial role in virus infection. Additionally, we have provided the first evidence of a connection between trypsin and a receptor, AaARLP, revealing its impact on enzymatic activity during infection. Our results represent a significant step toward understanding the metabolic pathways in mosquitoes and its impact to mosquito–virus interactions (Fig. 6). These findings could inform the development of targeted strategies for mosquito-borne disease control, such as designing novel interventions to disrupt this pathway and reduce vectorial capacity for managing mosquito-borne diseases.

## MATERIALS AND METHODS

### Mosquitoes

The *A. aegypti* mosquitoes (Orlando strain) were reared in a climate-controlled environment at 28°C with a relative humidity of 60%–80%, under a 14:10 h light-dark cycle. Larvae, hatched at an approximate density of 200 per pan, were fed with fish food (Wardley Aquatics). Adult mosquitoes had unrestricted access to cotton rolls soaked in a 10% sucrose solution. To sustain the mosquito colonies, female mosquitoes were provided blood meals sourced from naïve AG129 mice.

### Alignment and identity percentages between species

The protein sequence of AaARLP (AAEL014717), adiponectin receptors 1 and 2 of *H. sapiens* (Q96A54 and Q86V24), and *M. musculus* (Q91VH1 and Q8BQS5) were obtained, and both alignment and identity percentages were generated through UniProt (uniprot.org) (41).

### Virus preparation and *A. aegypti* infection

The ZIKV (MEX2-81 strain, accession number KX446950) was propagated in *Aedes albopictus* C6/36 cells, cultured in Dulbecco modified Eagle medium (DMEM) (Gibco) supplemented with 10% fetal bovine serum (FBS) and 1% penicillin/streptomycin (Invitrogen). Cultures were maintained at 30°C with 5% CO_2_.

For virus titer determination, a plaque assay was utilized. BHK-21 cells were cultured in DMEM with 10% FBS and 1% penicillin/streptomycin at 37°C with 5% CO_2_. Cells were seeded into a 24-well plate and exposed to a series of diluted virus samples for 1 h. After incubation, an overlayer medium (DMEM with 2% FBS, 2% carboxymethylcellulose, and 1% penicillin/streptomycin) was added and cultured for 5 days. Fixation was done with a 10% formaldehyde solution, followed by staining with crystal violet solution. Plaques were counted to calculate PFU.

Female *A. aegypti*, aged 4–7 days, were assigned to the naïve, control, or Zika group. The naïve group only had access to sucrose solution. The control group fed on a naïve AG129 mouse, while the Zika group fed on a mouse previously infected with ZIKV, having received a subcutaneous injection of 100 PFU 3 days before mosquito exposure. Mouse blood was collected simultaneously with mosquito infection. The blood sample was centrifuged at 2,000× *g* for 5 min, and the serum was collected. The serum was then processed using a plaque assay, as previously described, to determine the viral titer. After half an hour feeding, engorged females were collected, transferred to new containers, and provided with 10% sucrose solution *ad libitum* after a half-hour feeding period. The midgut was dissected at 1-, 2-, and 3-days post-feeding stages and stored at −80°C for RNA extraction.

### Gene silencing in Aag-2 and in *A. aegypti*

Double-stranded RNA (dsRNA) was designed to target either the green fluorescent protein (GFP) (Sequence template from pAc5.1B-EGFP, Addgene plasmid # 21181) (42) or the specific gene of interest [AaARLP (AAEL014717), late trypsin 1 (AAEL013284), trypsin (AAEL010196, AAEL013715), and trypsin 5G1 precursor (AAEL013712)]. The dsRNA template was generated using Polymerase Chain Reaction (PCR), following the protocol of Phusion High-Fidelity DNA Polymerase (New England Biolabs). This involved utilizing 400 µg of *A. aegypti* cDNA library from *A. aegypti* midgut or GFP plasmid in a 200 µL reaction with primers containing the T7 sequence (Table S2). PCR conditions comprised 1 cycle of 98°C for 30 s, followed by 40 cycles of 98°C for 10 s, 55°C for 30 s, and 72°C for 30 s, capped by 1 cycle of 72°C for 5 min. Subsequently, PCR products were purified using the Monarch DNA Gel Extraction Kit (New England Biolabs). The template used for dsRNA synthesis was generated with the TranscriptAid T7 High Yield Transcription Kit (Thermo Scientific). The reaction involved a 4-h incubation at 37°C, followed by the purification of RNA transcripts according to the manufacturer’s protocol. The concentration of dsRNA was measured using NanoDrop 2000C Spectrophotometers (Thermo Scientific).

The *A. aegypti* Aag-2 cells (43) were cultured in Schneider’s Drosophila Medium (Gibco) supplemented with 10% FBS and 1% penicillin/streptomycin (Invitrogen), maintaining them at 30°C with 5% CO_2_. Cells were seeded into 48-well plates overnight before transfection. Transfection was carried out with 500 ng of dsRNA per well using INTERFEin (Polyplus) according to the standard protocol (44). Three days post-transfection, cells were infected with 0.1 MOI of ZIKV and then incubated for 2 days. RNA was subsequently extracted from the cells using TRIzol Reagent (Thermo Fisher Scientific).

One hundred and twenty female *A. aegypti* aged 4–7 days were microinjected with 500 ng of dsGFP, dsAaARLP, ds3284, ds0196, ds3712, or ds3715, or 125 ng of each dsRNA for trypsin (ds3284, ds0196, ds3712 and ds3715) into dsMiX using the Nanoject II Auto-Nanoliter Injector (Drummond Scientific). After 3 days post-injection, injected mosquitoes were exposed to ZIKV-infected AG129 mice following the previously described procedure. The midgut was dissected after 2 and 7 days of infection, and individual midgut RNA was extracted using NucleoSpin RNA Columns (Takara Bio). The remaining body from day 7 was collected, and RNA was extracted using TRIzol Reagent (Thermo Fisher Scientific). The cDNA was generated with the iScript cDNA Synthesis Kit (Bio-Rad), and real-time PCR was used to quantify virus and gene expression levels with the reference gene RP49 (Table S2). The infection rate was calculated based on the presence of ZIKV infection in the midgut on day 7. Only the bodies with a positive midgut were included in the calculation of the dissemination rate. Analysis and figures only include samples with positive ZIKV infection.

### Transcriptome analyses

Female *A. aegypti* mosquitoes microinjected with dsRNA (dsGFP and ds*AaARLP*) were subsequently fed on ZIKV-infected AG129 mouse, as previously described. Two- and seven-days post-infection, the mosquitoes were collected for midgut dissection. Midgut RNA extraction followed the aforementioned procedure. Three midgut RNA samples from each group were then submitted for library preparation and sequenced using Illumina HiSeq 2500 through paired-end sequencing at the Yale Centre for Genome Analysis (YCGA). The transcript data were aligned to the *A. aegypti* LVP_AGWG AaegL5 transcripts reference files, available on VectorBase (https://vectorbase.org/) using HISAT 2 (45, 46). The counts generated were processed by DESeq2 (47) using Partek Flow software, v11.0. The transcriptomic data are available in the Sequence Read Archive (SRA) database at the NCBI with accession number PRJNA1140660. The functional enrichment analysis were performed from g:GOSt (g:Profiler version e111_eg58_p18_f463989d).

### Trypsin activity assay

The *A. aegypti* female mosquitoes microinjected with dsRNA (dsGFP and ds*AaARLP*) were subsequently fed on AG129 mice. At 24 h post-blood meal, midguts per group were dissected, collected in 50 µL of buffer solution (50 mM Tris-HCl, pH 8.0, with 10 mM CaCl_2_), with 10 midguts per tube, and homogenized on ice using a pestle. Supernatants were obtained following high-speed centrifugation (18,400 rcf) at 4°C. The trypsin activity assays reaction mixtures, each containing 5 µL of midgut extract and 200 µL 1 mM Na-benzoyl-D, L-arginine-p-nitroanilide hydrochloride (BApNA) (Sigma), were then incubated at 37°C for 5 min, and absorbance values were measured in a plate reader at A405nm (31, 32).

### Statistical analysis

Independent biological replicates were performed for each experiment. The technical replicates were applied in both plaque assay and real-time PCR. Statistical analyses were conducted using the Mann–Whitney test to compare between two groups or Tukey’s honestly significant difference (HSD) test for two-way ANOVA to perform multiple comparisons between groups or time points, employing GraphPad Prism software (Prism 10). Fisher’s exact test was used to analyze the *P*-value between infection and dissemination rate. A *P*-value of <0.05 was considered statistically significant.

## SUPPLEMENTAL MATERIAL

Supplemental material mbio.02433-24-s0001.docx Tables S1 and S2; Fig. S1 and S2.

ASM does not own the copyrights to Supplemental Material that may be linked to, or accessed through, an article. The authors have granted ASM a non-exclusive, world-wide license to publish the Supplemental Material files. Please contact the corresponding author directly for reuse.

## References

[1] Musso D, Gubler DJ. 2016. Zika virus. Clin Microbiol Rev 29:487–524. doi:10.1128/CMR.00072-15 27029595 PMC4861986

[2] Plourde AR, Bloch EM. 2016. A literature review of Zika virus. Emerg Infect Dis 22:1185–1192. doi:10.3201/eid2207.151990 27070380 PMC4918175

[3] Musso D, Ko AI, Baud D. 2019. Zika virus infection - after the pandemic. N Engl J Med 381:1444–1457. doi:10.1056/NEJMra1808246 31597021

[4] Ryan SJ, Carlson CJ, Mordecai EA, Johnson LR. 2019. Global expansion and redistribution of Aedes-borne virus transmission risk with climate change. PLoS Negl Trop Dis 13:e0007213. doi:10.1371/journal.pntd.0007213 30921321 PMC6438455

[5] Poland GA, Ovsyannikova IG, Kennedy RB. 2019. Zika vaccine development: current status. Mayo Clin Proc 94:2572–2586. doi:10.1016/j.mayocp.2019.05.016 31806107 PMC7094556

[6] Prince BC, Walsh E, Torres TZB, Rückert C. 2023. Recognition of arboviruses by the mosquito immune system. Biomolecules 13:1159. doi:10.3390/biom13071159 37509194 PMC10376960

[7] Sigle LT, McGraw EA. 2019. Expanding the canon: non-classical mosquito genes at the interface of arboviral infection. Insect Biochem Mol Biol 109:72–80. doi:10.1016/j.ibmb.2019.04.004 30970277

[8] Ratnayake OC, Chotiwan N, Saavedra-Rodriguez K, Perera R. 2023. The buzz in the field: the interaction between viruses, mosquitoes, and metabolism. Front Cell Infect Microbiol 13:1128577. doi:10.3389/fcimb.2023.1128577 37360524 PMC10289420

[9] Yamauchi T, Kamon J, Ito Y, Tsuchida A, Yokomizo T, Kita S, Sugiyama T, Miyagishi M, Hara K, Tsunoda M, et al.. 2003. Cloning of adiponectin receptors that mediate antidiabetic metabolic effects. Nature New Biol 423:762–769. doi:10.1038/nature01705 12802337

[10] Kadowaki T, Yamauchi T. 2005. Adiponectin and adiponectin receptors. Endocr Rev 26:439–451. doi:10.1210/er.2005-0005 15897298

[11] Okada-Iwabu M, Yamauchi T, Iwabu M, Honma T, Hamagami K, Matsuda K, Yamaguchi M, Tanabe H, Kimura-Someya T, Shirouzu M, Ogata H, Tokuyama K, Ueki K, Nagano T, Tanaka A, Yokoyama S, Kadowaki T. 2013. A small-molecule AdipoR agonist for type 2 diabetes and short life in obesity. Nat New Biol 503:493–499. doi:10.1038/nature12656 24172895

[12] Otvos L Jr, Knappe D, Hoffmann R, Kovalszky I, Olah J, Hewitson TD, Stawikowska R, Stawikowski M, Cudic P, Lin F, Wade JD, Surmacz E, Lovas S. 2014. Development of second generation peptides modulating cellular adiponectin receptor responses. Front Chem 2:93. doi:10.3389/fchem.2014.00093 25368867 PMC4201147

[13] Kadowaki T, Yamauchi T, Kubota N, Hara K, Ueki K, Tobe K. 2006. Adiponectin and adiponectin receptors in insulin resistance, diabetes, and the metabolic syndrome. J Clin Invest 116:1784–1792. doi:10.1172/JCI29126 16823476 PMC1483172

[14] Kwak S-J, Hong S-H, Bajracharya R, Yang S-Y, Lee K-S, Yu K. 2013. Drosophila adiponectin receptor in insulin producing cells regulates glucose and lipid metabolism by controlling insulin secretion. PLoS ONE 8:e68641. doi:10.1371/journal.pone.0068641 23874700 PMC3709998

[15] Chuang Y-M, Stone H, Abouneameh S, Tang X, Fikrig E. 2024. Signaling between mammalian adiponectin and a mosquito adiponectin receptor reduces Plasmodium transmission. MBio 15:e0225723. doi:10.1128/mbio.02257-23 38078744 PMC10790699

[16] Tang X, Cao Y, Arora G, Hwang J, Sajid A, Brown CL, Mehta S, Marín-López A, Chuang Y-M, Wu M-J, Ma H, Pal U, Narasimhan S, Fikrig E. 2021. The lyme disease agent co-opts adiponectin receptor-mediated signaling in its arthropod vector. Elife 10:e72568. doi:10.7554/eLife.72568 34783654 PMC8639152

[17] Roy B, Palaniyandi SS. 2021. Tissue-specific role and associated downstream signaling pathways of adiponectin. Cell Biosci 11:77. doi:10.1186/s13578-021-00587-4 33902691 PMC8073961

[18] Trammell CE, Ramirez G, Sanchez-Vargas I, St Clair LA, Ratnayake OC, Luckhart S, Perera R, Goodman AG. 2022. Coupled small molecules target RNA interference and JAK/STAT signaling to reduce Zika virus infection in Aedes aegypti. PLoS Pathog 18:e1010411. doi:10.1371/journal.ppat.1010411 35377915 PMC9017935

[19] Noriega FG, Wells MA. 1999. A molecular view of trypsin synthesis in the midgut of Aedes aegypti*.* J Insect Physiol 45:613–620. doi:10.1016/s0022-1910(99)00052-9 12770346

[20] Toprak U. 2020. The role of peptide hormones in insect lipid metabolism. Front Physiol 11:434. doi:10.3389/fphys.2020.00434 32457651 PMC7221030

[21] Tang YT, Hu T, Arterburn M, Boyle B, Bright JM, Emtage PC, Funk WD. 2005. PAQR proteins: a novel membrane receptor family defined by an ancient 7-transmembrane pass motif. J Mol Evol 61:372–380. doi:10.1007/s00239-004-0375-2 16044242

[22] Ruan H, Dong LQ. 2016. Adiponectin signaling and function in insulin target tissues. J Mol Cell Biol 8:101–109. doi:10.1093/jmcb/mjw014 26993044 PMC4816150

[23] Bonizzoni M, Dunn WA, Campbell CL, Olson KE, Dimon MT, Marinotti O, James AA. 2011. RNA-seq analyses of blood-induced changes in gene expression in the mosquito vector species, Aedes aegypti. BMC Genomics 12:1–13. doi:10.1186/1471-2164-12-82 PMC3042412 21276245

[24] El Safadi D, Lebeau G, Turpin J, Lefebvre d’Hellencourt C, Diotel N, Viranaicken W, Krejbich-Trotot P. 2023. The antiviral potential of adiporon, an adiponectin receptor agonist, reveals the ability of Zika virus to deregulate adiponectin receptor expression. Viruses 16:24. doi:10.3390/v16010024 38257725 PMC10820441

[25] Pennington J, Noriega F, Wells M. 1995. The expression of early trypsin in *Aedes-aegypti*, p 211–211. In Wiley-liss div john wiley & sons inc 605 third ave. NEW YORK.

[26] Noriega FG, Shah DK, Wells MA. 1997. Juvenile hormone controls early trypsin gene transcription in the midgut of Aedes aegypti*.* Insect Mol Biol 6:63–66. doi:10.1046/j.1365-2583.1997.00154.x 9013256

[27] Graf R, Briegel H. 1985. Isolation of trypsin isozymes from the mosquito Aedes aegypti (L.). Insect Biochem 15:611–618. doi:10.1016/0020-1790(85)90122-2

[28] Graf R, Raikhel AS, Brown MR, Lea AO, Briegel H. 1986. Mosquito trypsin: immunocytochemical localization in the midgut of blood-fed Aedes aegypti (L.). Cell Tissue Res 245:19–27. doi:10.1007/BF00218082 3524850

[29] Barillas-Mury C, Graf R, Hagedorn HH, Wells MA. 1991. cDNA and deduced amino acid sequence of a blood meal-induced trypsin from the mosquito, Aedes aegypti. Insect Biochem 21:825–831. doi:10.1016/0020-1790(91)90089-W

[30] Molina-cruz A, Gupta L, Richardson J, Bennett K, Black W, Barillas-Mury C. 2005. Effect of mosquito midgut trypsin activity on dengue-2 virus infection and dissemination in Aedes aegypti*.* Am J Trop Med Hyg 72:631–637. doi:10.4269/ajtmh.2005.72.631 15891140

[31] Brackney DE, Foy BD, Olson KE. 2008. The effects of midgut serine proteases on dengue virus type 2 infectivity of Aedes aegypti. Am J Trop Med Hyg 79:267–274. 18689635 PMC2745300

[32] Angleró-Rodríguez YI, Talyuli OA, Blumberg BJ, Kang S, Demby C, Shields A, Carlson J, Jupatanakul N, Dimopoulos G. 2017. An Aedes aegypti-associated fungus increases susceptibility to dengue virus by modulating gut trypsin activity. Elife 6:e28844. doi:10.7554/eLife.28844 29205153 PMC5716662

[33] Mao X, Kikani CK, Riojas RA, Langlais P, Wang L, Ramos FJ, Fang Q, Christ-Roberts CY, Hong JY, Kim R-Y, Liu F, Dong LQ. 2006. APPL1 binds to adiponectin receptors and mediates adiponectin signalling and function. Nat Cell Biol 8:516–523. doi:10.1038/ncb1404 16622416

[34] Xi Z, Ramirez JL, Dimopoulos G. 2008. The Aedes aegypti toll pathway controls dengue virus infection. PLoS Pathog 4:e1000098. doi:10.1371/journal.ppat.1000098 18604274 PMC2435278

[35] Kramer LD, Ciota AT. 2015. Dissecting vectorial capacity for mosquito-borne viruses. Curr Opin Virol 15:112–118. doi:10.1016/j.coviro.2015.10.003 26569343 PMC4688158

[36] Rückert C, Ebel GD. 2018. How do virus-mosquito interactions lead to viral emergence? Trends Parasitol 34:310–321. doi:10.1016/j.pt.2017.12.004 29305089 PMC5879000

[37] Zeledon EV, Baxt LA, Khan TA, Michino M, Miller M, Huggins DJ, Jiang CS, Vosshall LB, Duvall LB. 2024. Next-generation neuropeptide Y receptor small-molecule agonists inhibit mosquito-biting behavior. Parasites Vectors 17:276. doi:10.1186/s13071-024-06347-w 38937807 PMC11212260

[38] Chen T-Y, Smartt CT. 2021. Activation of the autophagy pathway decreases dengue virus infection in Aedes aegypti cells. Parasites Vectors 14:551. doi:10.1186/s13071-021-05066-w 34702321 PMC8549150

[39] Hixson B, Taracena ML, Buchon N. 2021. Midgut epithelial dynamics are central to mosquitoes’ physiology and fitness, and to the transmission of vector-borne disease. Front Cell Infect Microbiol 11:653156. doi:10.3389/fcimb.2021.653156 33842397 PMC8027260

[40] Chen T-Y, Raduwan H, Marín-López A, Cui Y, Fikrig E. 2024. Zika virus exists in enterocytes and enteroendocrine cells of the Aedes aegypti midgut. i Sci 27:110353. doi:10.1016/j.isci.2024.110353 PMC11269924 39055935

[41] The UniProt Consortium. 2023. UniProt: the universal protein knowledgebase in 2023. Nucleic Acids Res 51:D523–D531. 36408920 10.1093/nar/gkac1052 PMC9825514

[42] Tritschler F, Eulalio A, Truffault V, Hartmann MD, Helms S, Schmidt S, Coles M, Izaurralde E, Weichenrieder O. 2007. A divergent Sm fold in EDC3 proteins mediates DCP1 binding and P-body targeting. Mol Cell Biol 27:8600–8611. doi:10.1128/MCB.01506-07 17923697 PMC2169425

[43] Barletta ABF, Silva M, Sorgine MHF. 2012. Validation of Aedes aegypti Aag-2 cells as a model for insect immune studies. Parasites Vectors 5:1–9. doi:10.1186/1756-3305-5-148 22827926 PMC3419682

[44] Marin-Lopez A, Jiang J, Wang Y, Cao Y, MacNeil T, Hastings AK, Fikrig E. 2021. Aedes aegypti SNAP and a calcium transporter ATPase influence dengue virus dissemination. PLoS Negl Trop Dis 15:e0009442. doi:10.1371/journal.pntd.0009442 34115766 PMC8195420

[45] Kim D, Langmead B, Salzberg SL. 2015. HISAT: a fast spliced aligner with low memory requirements. Nat Methods 12:357–360. doi:10.1038/nmeth.3317 25751142 PMC4655817

[46] Kim D, Paggi JM, Park C, Bennett C, Salzberg SL. 2019. Graph-based genome alignment and genotyping with HISAT2 and HISAT-genotype. Nat Biotechnol 37:907–915. doi:10.1038/s41587-019-0201-4 31375807 PMC7605509

[47] Love MI, Huber W, Anders S. 2014. Moderated estimation of fold change and dispersion for RNA-seq data with DESeq2. Genome Biol 15:550. doi:10.1186/s13059-014-0550-8 25516281 PMC4302049

