# Supplementary material for: Aedes aegypti adiponectin receptor-like protein signaling facilitates Zika virus infection
Source: mBio. 2024 Oct 7;15(11):e02433-24. doi: 10.1128/mbio.02433-24 (PMC11559040; doi:10.1128/mbio.02433-24)
Supplement: Supplemental material — Tables S1 and S2; Fig. S1 and S2. [file mbio.02433-24-s0001.docx]

**Supplementary information**

**Table S1. RNA-seq results comparing gene expression in dsGFP and ds*AaARLP* *Ae. aegypti* midgut at 7 days post-infection.** Genes with significant differences are listed based on criteria of a p-value < 0.05, false discovery rate ≤ 0.1, and a fold change > 2.

| **Gene ID** | **Gene name** | **p-value** | **Fold change** |
| --- | --- | --- | --- |
| AAEL010196 | trypsin | 3.35E-21 | 1.13E+02 |
| AAEL013712 | trypsin 5G1 Precursor | 6.38E-15 | 1.13E+02 |
| AAEL013715 | trypsin | 1.26E-14 | 1.11E+02 |
| AAEL023729 |  | 8.71E-28 | 4.88E+01 |
| AAEL013284 | late trypsin 1, serine-type enodpeptidase | 1.53E-10 | 4.52E+01 |
| AAEL010202 | trypsin | 1.75E-05 | 2.54E+01 |
| AAEL014717 | adiponectin receptor | 3.21E-04 | 1.48E+01 |
| AAEL001693 | serine-type enodpeptidase | 7.25E-10 | 4.76E+00 |
| AAEL002263 |  | 7.74E-05 | 4.39E+00 |
| AAEL000859 |  | 1.66E-05 | 4.33E+00 |
| AAEL018241 |  | 1.21E-04 | 3.90E+00 |
| AAEL008080 | trypsin-eta, putative | 7.23E-08 | 3.85E+00 |
| AAEL002263 |  | 1.11E-04 | 3.84E+00 |
| AAEL003424 | zinc carboxypeptidase | 1.20E-04 | 3.34E+00 |
| AAEL010782 | carboxypeptidase | 3.13E-07 | 3.20E+00 |
| AAEL000746 | NADP-specific isocitrate dehydrogenase | 2.87E-05 | 3.19E+00 |
| AAEL008600 | zinc carboxypeptidase | 2.65E-04 | 2.70E+00 |
| AAEL005536 | tetraspanin 29fb | 3.91E-04 | 2.61E+00 |
| AAEL003066 | brain chitinase and chia | 2.20E-04 | 2.61E+00 |
| AAEL012845 | Mitochondrial import inner membrane translocase subunit TIM44 | 3.93E-04 | -4.87E+00 |
| AAEL011371 |  | 1.98E-04 | -5.17E+00 |
| AAEL005536 | tetraspanin 29fb | 2.46E-06 | -5.53E+00 |

**Table S2. Primer list for dsRNA and Real-time PCR.**

|  | Sequence (5’-3’) |
| --- | --- |
| F-T7-GFP | TAATACGACTCACTATAGGGACGTAAACGGCCACAAGTTC |
| R-T7-GFP | TAATACGACTCACTATAGGGTGTTCTGCTGGTAGTGGTCG |
| F-T7-AaARLP | TAATACGACTCACTATAGGGGCCTTCGTGCACTACCACGGC |
| R-T7-AaARLP | TAATACGACTCACTATAGGGCTTTCTCGTGTAGATGCAATGG |
| F-T7-3284 | TAATACGACTCACTATAGGGTAAACGGACAAACGGCTACC |
| R-T7-3284 | TAATACGACTCACTATAGGGCTGTAGGCCTTCGAGCATTC |
| F-T7-0196 | TAATACGACTCACTATAGGGTTCTGGCCAACCAAATCTTC |
| R-T7-0196 | TAATACGACTCACTATAGGGGCTCAAGTAAGCCTTGTGGC |
| F-T7-3712 | TAATACGACTCACTATAGGGTTGGATCTTCCCATTTCTGC |
| R-T7-3712 | TAATACGACTCACTATAGGGGCTCAAGTAAGCCTTGTGGC |
| F-T7-3715 | TAATACGACTCACTATAGGGGCGGTTACGAAGTGGACATT |
| R-T7-3715 | TAATACGACTCACTATAGGGAAAACCAGCGCATAACATCC |
| F-Zika | TTGGTCATGATACTGCTGATTGC |
| R-Zika | CCTTCCACAAAGTCCCTATTGC |
| F-AaARLP | CCGCTCCCAGACGATGATTT |
| R-AaARLP | TAGGGAATCGTCCTCCTCCG |
| F-3284 | ACAGTACCAGTATTCGGCAAA |
| R-3284 | GAGAACTTGGAATGGGAACT |
| F-0196 | CGCATTGTTGGTGGCTTTGA |
| R-0196 | ACCCAGCGTTCGGAAAGTAG |
| F-3712 | CCGACTACGATTTTGCGCTG |
| R-3712 | TGAGTATTACCCCAGCCGGA |
| F-3715 | TTGGATTGGTGACTCCACGG |
| R-3715 | TAACCTTCCTCGGCACAACC |
| F-RP49 | GCTATGACAAGCTTGCCCCCA |
| R-RP49 | TCATCAGCACCTCCAGCT |

**
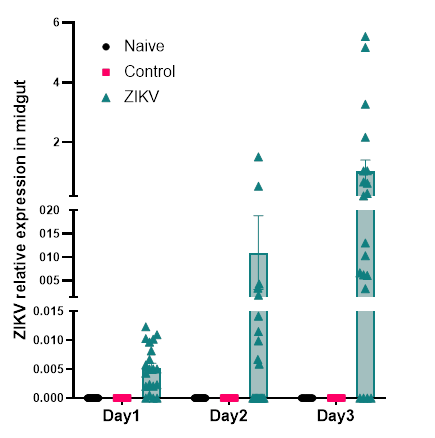
**

**Fig. S1. First three days of Zika virus expression level in *Ae. aegypti* midgut.**

Zika virus in the Ae. aegypti midgut among Naïve, Control, and ZIKV groups at different time points. Each dot represents one midgut sample, with error bars indicating the standard error of the mean (SEM). Day 1: Naïve N=19, Control N=20, and ZIKV N=18; Day 2: Naïve N=18, Control N=14, and ZIKV N=16; Day 3: Naïve N=18, Control N=20, and ZIKV N=20.


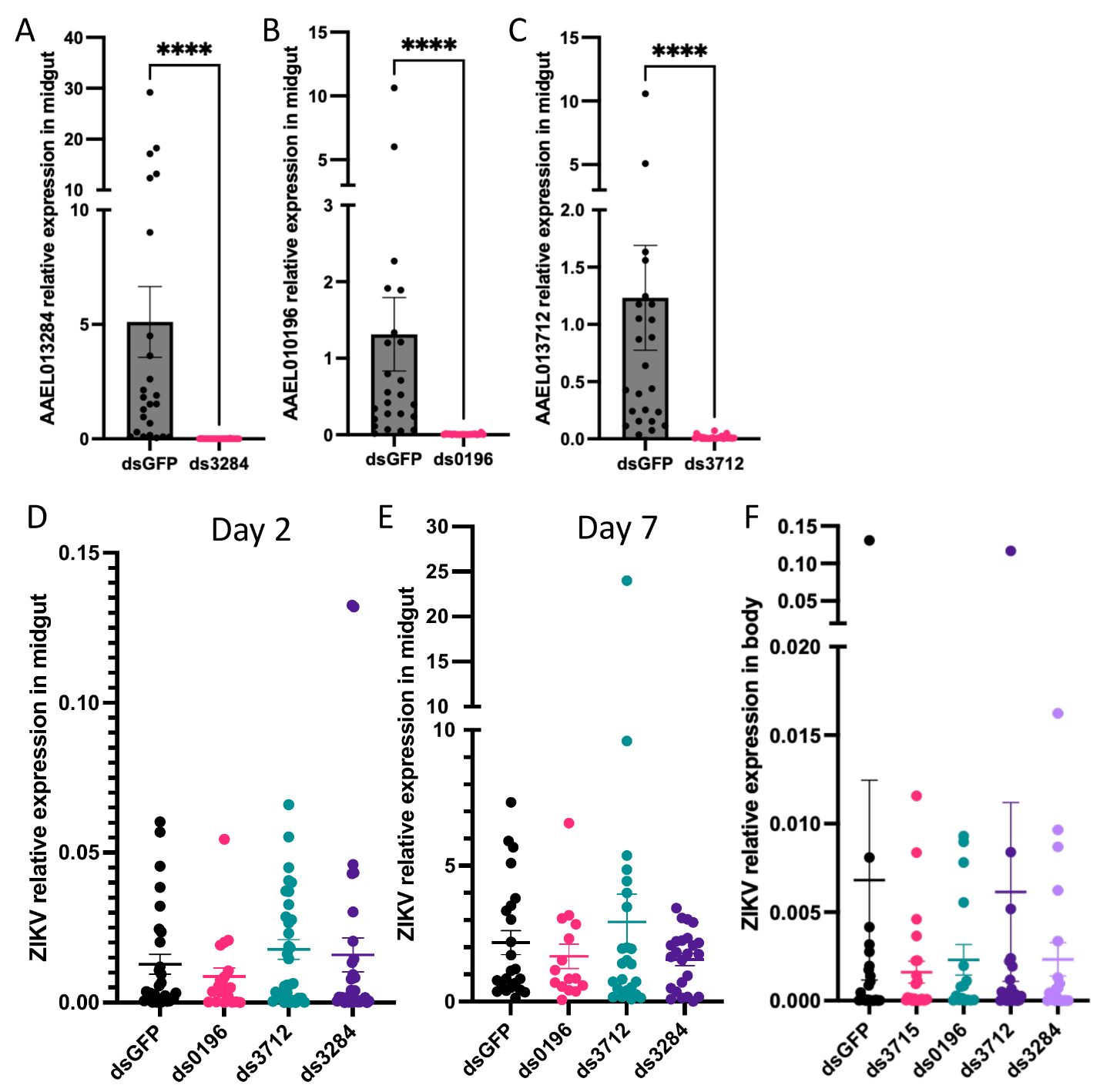


**Fig. S2. Impact of single trypsin silencing on Zika virus infection in *Ae. aegypti*.** (A) Relative expression levels of trypsin (AAEL013284) between dsGFP and ds3284, (B) relative expression levels of trypsin (AAEL010196) between dsGFP and ds0196, and (C) relative expression levels of trypsin (AAEL013712) between dsGFP and ds3712 at 2 days post-infection in the *Ae. aegypti* midgut. ZIKV RNA copy numbers in different dsRNA microinjected *Ae. aegypti* at (D) 2 days in midgut, (E) 7 days in midgut, and (F) 7 days in the body post-ZIKV infection. Each dot represents one midgut sample, with error bars indicating the standard error of the mean (SEM). Day 2 Midgut: dsGFP N=29, ds0196 N=20, ds3712 N=33, and ds3284 N=33; Day 7 Midgut: dsGFP N=23, ds0196 N=15, ds3712 N=24, and ds3284 N=24; Day 7 body: dsGFP N=23, ds3715 N= 23, ds0196 N=15, ds3712 N=21, and ds3284 N=23.**** p-value < 0.0001.
